# Synchronous Acute Appendicitis and Acute Cholecystitis: A Case Report

**DOI:** 10.7759/cureus.40411

**Published:** 2023-06-14

**Authors:** Mihaela Kancheva, Vladimir Neychev

**Affiliations:** 1 Surgery, University of Central Florida College of Medicine, Orlando, USA

**Keywords:** right lower quadrant pain, right upper quadrant pain, right flank pain, cholecystitis, appendicitis

## Abstract

Acute cholecystitis (AC) and acute appendicitis (AA), independently, are among the most commonly diagnosed conditions in the emergency department (ED). However, their synchronous presentation is very rare. Here, we present a 31-year-old man with worsening right flank abdominal pain, nausea, and vomiting. Physical examination results were significant for moderate to severe right upper abdominal quadrant pain with a positive Murphy’s sign and right lower quadrant pain with rebound. Workup in the ED revealed leukocytosis with a left shift, and the abdominal ultrasound and computerized tomography scan showed AA and AC. A literature review revealed a paucity of publications on concomitant AA and AC. Reporting new cases will contribute to improving our understanding of the biology, natural history, and management of this rare pathological combination.

## Introduction

Acute appendicitis (AA) and acute cholecystitis (AC), independently, are common diagnosed conditions, with a yearly estimate of approximately 350,000 AA and 200,000 AC cases diagnosed in the United States [1,2]. Individually, their presentations are well studied. Common symptoms of AA include pain in the epigastrium that migrates to the right lower quadrant (RLQ) and may present with severe pain and punctum maximum at McBurney’s point [3-5]. Patients with AC tend to present with pain in the right upper quadrant (RUQ) or epigastrium with prolonged and mild to severe pain, possible radiation to the right shoulder or back, and Murphy’s sign [6]. Patients with AA or AC can also present with fever, nausea/vomiting, and anorexia [6,7].

Separately diagnosing AC and AA is straightforward and usually supported and confirmed by imaging technologies, including ultrasound, commonly used for the initial diagnosis of AC, and computerized tomography (CT), most often performed for possible AA [1,3,6,7]. Laparoscopic surgery is the gold-standard treatment for both conditions [1,8]. However, only a few cases of the synchronous presentation of AA and AC have been reported. Here, we present a rare case of simultaneous AA and AC and provide a comprehensive review of cases, outlining the common presenting features of a patient with both conditions.

## Case presentation

A 31-year-old otherwise healthy man presented to the emergency department (ED) reporting worsening right flank abdominal pain over the past several days, associated with nausea, vomiting, and fever. The patient denied any medical, surgical, or known family health history. He stated that the pain was severe and mainly in the RUQ and RLQ.

The patient's physical examination findings are significant for a body temperature of 38.4 °C (101.2 °F), heart rate of 103 bpm, respiratory rate of 18, oxygen saturation (SpO_2_) of 98%, and blood pressure of 138/81 mmHg. He had a non-distended abdomen with moderate to severe tenderness in the RUQ with a positive Murphy's sign and in the RLQ with punctum maximum at McBurney's point. The remainder of the physical examination results were unremarkable.

The workup in the ED, including complete blood count and complete metabolic panel, showed an elevated white blood cell count of 12.17 10*3/uL with a left shift, elevated aspartate transferase of 91 U/L, alanine transaminase of 119 U/L, and glucose of 119 mg/dL. The urinalysis results were within normal limits.

**Figure 1:**
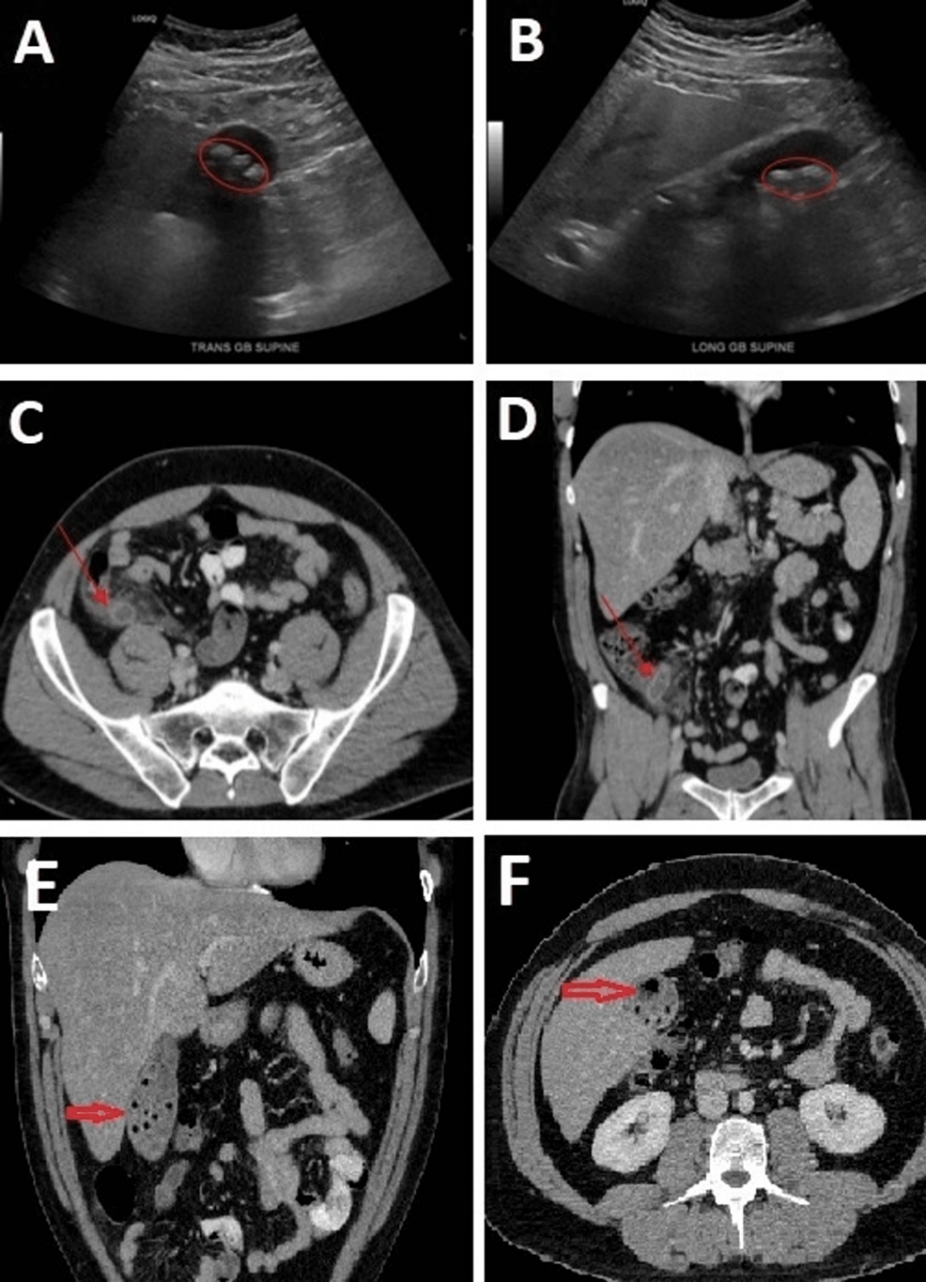
A - transverse and B - longitudinal ultrasound views of the liver and gallbladder with outlined gallbladder stones and thickened edematous wall; C – axial and D – coronal contrasted CT scan views of the abdomen and pelvis with an arrow pointing at an inflamed and distended appendix with parapedicular stranding and edema; E – coronal and F – axial contrasted CT scan images depicting a distended gallbladder with the red arrow pointing to the hyperemic wall and multiple gallstones in panel E and with the red arrow pointing to a small pocket of air, suggesting empyema of the gallbladder in panel F.

An abdominal ultrasound and CT scan with IV contrast of the abdomen and pelvis were performed in the ED, revealing concurrent AC (Figures 1A, 1B, 1E, 1F) and incidental AA (Figures 1C, 1D). A distended gallbladder with multiple gallstones, a hyperemic wall, and a small pocket of air, suggestive of empyema, was also depicted in the contrasted CT scans (Figures 1E, 1F). Preoperatively, the patient was resuscitated with IV fluids and started on empiric IV antibiotics, specifically 4.5 g piperacillin/tazobactam, every eight hours for the two-day hospital stay to maximize active excretion through the biliary tract and broad-spectrum coverage including gram-positive, gram-negative, and anaerobic microorganisms. IV pain control and antiemetics were also administered. A detailed discussion was carried out with the patient and his family about the biology, natural history, and management options of the simultaneously identified AC and AA. Altogether, emergent laparoscopic cholecystectomy and appendectomy were advised, and the patient agreed to proceed with the procedure.

Access to the peritoneal cavity was gained with a 5 mm OPTIVIEW® laparoscopic trocar (Ethicon, Inc., Raritan, New Jersey, United States) at the Palmer’s point. After establishing pneumoperitoneum, three additional 5 mm trocars were placed (one 2 cm above the umbilicus and two 3 cm below the right subcostal margin along the middle and posterior axillary lines). The 5 mm OPTIVIEW® laparoscopic trocar was upgraded to a 12 mm port. The patient was initially positioned in Fowler's position with the right side up in preparation for laparoscopic cholecystectomy. Significant distention of the gallbladder was noted, and the drainage with an endoscopic aspiration needle revealed empyema/abscess of the gallbladder.

After decompressing the gallbladder, an uneventful laparoscopic cholecystectomy was performed, and the gallbladder was sent to pathology as a permanent specimen. The same port access sites were used for the appendectomy portion. The patient was repositioned in the Trendelenburg position with the right side up. Significant inflammatory changes in the RLQ were caused by gangrenous appendicitis with local serous-fibrinous periappendicitis. An uneventful appendectomy was performed, and a specimen was sent to pathology as a permanent specimen. The final surgical pathology report was consistent with acute on chronic cholecystitis with cholelithiasis and gangrenous appendicitis.

The patient tolerated and recovered from the procedure well. He was discharged home on postoperative day two without post-surgical complications, with well-controlled, mild post-surgical pain at the port site incisions, tolerating a regular diet, and passing flatus and bowel movement. As expected, the patient had a follow-up visit in the outpatient surgery clinic three weeks after the procedure with an uneventful recovery course.

## Discussion

The classic presentations of AA and AC have been extensively described in the literature; however, patients with AA and AC do not always present with classic symptoms that may lead to diagnostic and management challenges [3-6,9]. The rare cases of synchronous AA and AC with combined and less defined symptoms and features, such as in the patient presented here, may contribute to the diagnostic and management deception. We reviewed previously published cases, compared them to ours, and summarized the data in Table 1 with more comprehensive information, including the initial presentation, diagnostic investigations, physical examinations, and treatment approaches [10].

**Table 1:** Cases of concurrent AA and AA. AA: acute appendicitis; Abd: abdomen; AC: acute cholecystitis; Alk P: alkaline phosphatase; AST: aspartate aminotransferase; F: female; HR: heart rate; Lap A&C: laparoscopic appendectomy and laparoscopic cholecystectomy; M: male; N/V: nausea and non-bilious, non-bloody vomiting; PTGBD: performed sono-guide percutaneous transhepatic gallbladder drainage; RLQ: right lower quadrant; RS: right side; RUQ: right upper quadrant; T: temperature; TB: total bilirubin; WBC: white blood cell

Sex	Age	Presenting complaints	Positive physical exam findings	Abnormal vital signs	Abnormal labs	Procedure and pathology
M	23	12-hour severe RUQ and RLQ pain began in the epigastrium with N/V and anorexia.	Tenderness in the RUQ and right iliac fossa, guarding, and rebound tenderness	T: 38.5 °C	WBC: 14.3 x 109/L; TB: 54 μmol/L; AST: 39 U/L	Lap A&C showing AA and AC without gallstones. The gallbladder wall also had diffuse inflammation, edema, necrosis, and extensive venous thrombi [11].
F	29	24-hour progressive RS Abd pain in the RLQ with subcostal pain and N/V	Tenderness to palpation; Murphy’s sign; McBurney’s point	Reported as normal	WBC: 13.3 B/L	Diagnostic Lap A&C revealing AA and AC. Confirmed on the pathology report [12].
F	36	Two-day severe, sharp RUQ pain radiating to the back	Epigastric tenderness; Murphy’s sign	Reported as normal	Reported as normal	Diagnostic laparoscopy leading to Lap A&C. Pathology confirmed calculous AC and AA [13].
M	40	18-hour N/V, and Abd pain localized most severely at the RUQ and RLQ. Original epigastric discomfort migrated to the RLQ.	Lethargy; scleral icterus; diffuse tenderness in Abd with rebound and guarding; Murphy’s sign; Rovsing’s sign; mild Abd distention	T: 102.5 °F; BP: 92/50 mmHg; HR: 115 bpm	TB: 2.7 mg/dL; Alk P: 178 IU/L	Lap A&C found severe AA and acute on chronic cholecystitis with mucosal congestion [14].
F	43	11-day RUQ pain with intermittent radiation to the RS	RS pain, Murphy sign	HR: 100 bpm; T: 38.5°C	WBC: 16,200/mm; TB 2.32 mg/dl	Utilized a Masson incision for a subtotal anterograde cholecystectomy and removal of a subhepatic abscess. Anterograde appendectomy was performed to remove a plastron and sub-serous retrocecal appendix [15].
F	45	One-day severe and sharp epigastric, RUQ, and RLQ pain with N/V	Epigastric tenderness; Murphy’s sign; tenderness over McBurney’s point	Reported as normal	TB: 1.4 mg/dL	Lap A&C found acute and chronic cholecystitis with cholelithiasis and non-perforated AA with periappendicitis [16].
F	66	Four-day RS Abd pain starting in the epigastrium, with increasing severity for 10 hours with nausea, fever, and tachycardia	Tenderness in the RS Abd (right iliac fossa); Murphy sign	HR: 105 bpm; T: 38.4°C	Reported as normal	Lap C and open appendectomy with intraoperative perforation of the appendix [17].
F	67	Two-day of central Abd pain migrated to the RUQ and RLQ with nausea and anorexia	Tenderness to percussion over RUQ and right iliac fossa	HR: 100 bpm	WBC: 18.5 x 10^9^/L	Lap A&C and found transmural acute inflammatory exudate of the appendix and gallbladder. Appendix also had ulceration of mucosa [18].
M	78	One-week sharp Abd pain with nausea, localized to the RUQ and RLQ, especially after meal, and radiated to the back	Significant local tenderness over the right abdomen, including epigastric pain; Murphy’s sign; obturator sign; tenderness over McBurney’s point	T: 37.8 °C	TB: 4.7 mg/dL; Alk P: 290 U/L	PTGBD for drainage of the bile juice and medically managed patient until Lap C. Non-surgical management of appendicitis due to patient refusal of Lap A. Diagnosis was AC with cholelithiasis, acute pancreatitis, and AA [19].

We found similarities and differences in the patients’ presentations compared to our case. The patients diagnosed with concurrent AA and AC primarily presented with right-sided pain - usually both RUQ and RLQ - and almost always with concurrent nausea and non-bilious vomiting. Physical examination results typically showed tenderness to palpation and Murphy’s sign. Many of the physical examinations had a positive McBurney’s point in the patient, and CT scans and ultrasound confirmed the combined AA and AC diagnosis, hence suggesting laparoscopic procedures in the patients [11-19]. This result is consistent with well-documented common symptoms of AA, which include RLQ pain, and common symptoms of AC, which include pain in the severe RUQ or epigastrium pain and possible radiation to the right shoulder or back [6,7].

Murphy’s sign is commonly seen as a diagnostic feature in the case studies of concurrent AA and AC, which is consistent with the findings of one study showing the sensitivity and specificity of Murphy’s sign in AC as 97% and 48%, respectively [9]. However, unlike in the case of combined AA and AC, McBurney's point is only seen in about 50% of patients with isolated AA [3]. The duration of symptoms and abnormalities in vital signs and laboratory results were inconsistent and hence are not a reliable basis of diagnosis in these patients [11-19]. Ultrasound is the most often used imaging modality for the initial diagnosis of AC, although CT and MRI are often also performed for possible complications, and laparoscopic cholecystectomy is the gold-standard treatment [1,3,6-8].

CT is the most commonly used imaging technique in diagnosing suspected AA, and laparoscopic appendectomy remains the first-line treatment in most cases [20]. Our patient had a similar presentation and synchronous AA and AC diagnosis, confirmed with ultrasound and CT scan. Similar to our case, simultaneous laparoscopic cholecystectomy and appendectomy are the treatments of choice in the majority of the reported cases; however, open surgery and medical management have been described in some cases [15,17,19].

## Conclusions

Considering concurrent AA and AC in patients presenting with generalized right-sided abdominal pain, regardless of the length of symptoms upon presentation, is imperative. A thorough medical history, physical examination, and appropriate imaging will reveal the concurrent pathology. Reporting cases of combined AA and AC will help us better understand the biology and natural history and help practitioners of all specialties recognize this rare condition.

## References

[1] Ferris M, Quan S, Kaplan BS (2017). The global incidence of appendicitis: a systematic review of population-based studies. Ann Surg.

[2] Wadhwa V, Jobanputra Y, Garg SK, Patwardhan S, Mehta D, Sanaka MR (2017). Nationwide trends of hospital admissions for acute cholecystitis in the United States. Gastroenterol Rep.

[3] Humes DJ, Simpson J (2012). Clinical presentation of acute appendicitis: clinical signs—laboratory findings—clinical scores, Alvarado score and derivate scores. Imaging of Acute Appendicitis in Adults and Children.

[4] Birnbaum BA, Wilson SR (2000). Appendicitis at the millennium. Radiology.

[5] Chung CH, Ng CP, Lai KK (2000). Delays by patients, emergency physicians, and surgeons in the management of acute appendicitis: retrospective study. Hong Kong Med J.

[6] Zakko SF, Afdhal NH (2023). Acute calculous cholecystitis: clinical features and diagnosis. UpToDate.

[7] Martin RF (2023). Acute appendicitis in adults: clinical manifestations and differential diagnosis. UpToDate.

[8] Katabathina VS, Zafar AM, Suri R (2015). Clinical presentation, imaging, and management of acute cholecystitis. Tech Vasc Interv Radiol.

[9] Singer AJ, McCracken G, Henry MC, Thode HC, Jr. Jr., Cabahug CJ (1996). Correlation among clinical, laboratory, and hepatobiliary scanning findings in patients with suspected acute cholecystitis. Ann Emerg Med.

[10] Buhamed F, Edward M, Shuaib A (2019). Synchronous acute appendicitis and acute cholecystitis, is it a myth or reality? A literature review. Open Access Emerg Med.

[11] Sahebally SM, Burke JP, Nolan N, Latif A (2011). Synchronous presentation of acute acalculous cholecystitis and appendicitis: a case report. J Med Case Rep.

[12] Shweiki E, Price TP, Patel PH (2016). Synchronous acute appendicitis and acute cholecystitis: a discussion of a century's worth of epidemiologic, basic science, and clinical research, explicating the pathophysiology of a likely underrecognized historical condition. Am Surg.

[13] Alkhurmudi M, Ali B, Alzaharani A (2022). Incidental finding of acute appendicitis during laparoscopic cholecystectomy for an acute calculous cholecystitis. Cureus.

[14] Victory J, Meytes V, Parizh D, Ferzli G, Nemr R (2017). Co-existent appendicitis and cholecystitis. Ann Laparosc Endosc Surg.

[15] Padrón-Arredondo G, de Atocha Rosado-Montero M (2016). [Synchronous acute cholecystolithiasis and perforated acute appendicitis. Case report]. Cir Cir.

[16] Demuro JP (2012). Simultaneous acute cholecystitis and acute appendicitis treated by a single laparoscopic operation. Case Rep Surg.

[17] Salih AM, Kakamad F, Abbas MH (2016). Acute cholecystitis with perforated appendicitis: the first reported case. J Case Rep Images Surg.

[18] Gandhi J, Tan J (2015). Concurrent presentation of appendicitis and acute cholecystitis: diagnosis of rare occurrence. BMJ Case Rep.

[19] Lee T-Y, Chang H-M, Shih M-L (2014). Successful nonsurgical treatment for synchronous acute cholecystitis and acute appendicitis: a case report and review of the literatures. J Med Sci.

[20] Moris D, Paulson EK, Pappas TN (2021). Diagnosis and management of acute appendicitis in adults: a review. JAMA.

